# Human umbilical cord matrix-derived stem cells expressing interferon-β gene inhibit breast cancer cells via apoptosis

**DOI:** 10.18632/oncotarget.8997

**Published:** 2016-04-26

**Authors:** Ching-Ju Shen, Te-Fu Chan, Chien-Chung Chen, Yi-Chiang Hsu, Cheng-Yu Long, Chung-Sheng Lai

**Affiliations:** ^1^ Graduate Institute of Medicine, College of Medicine, Kaohsiung Medical University, Kaohsiung, Taiwan; ^2^ Department of Obstetrics and Gynecology, Kaohsiung Medical University Hospital, Kaohsiung Medical University, Kaohsiung, Taiwan; ^3^ Department of Plastic and Reconstruction Surgery, E-Da Hospital, Taiwan; ^4^ Graduate Institute of Medical Science, College of Health Sciences, Chang Jung Christian University, Tainan, Taiwan; ^5^ Innovative Research Center of Medicine, College of Health Sciences, Chang Jung Christian University, Tainan, Taiwan; ^6^ Division of Plastic Surgery, Department of Surgery, Kaohsiung Medical University Hospital, Kaohsiung Medical University, Kaohsiung, Taiwan

**Keywords:** breast carcinoma, MDA-MB-231 cells, Hs578T cells, interferon-β, stem cell therapy

## Abstract

Human umbilical cord mesenchymal stem cells (hUCMSCs) derived from the umbilical cord matrix have been reported to be used as anti-tumor gene carrier for attenuation of tumor growth, which extends the half-life and lowers the unexpected cytotoxicity of the gene *in vivo*. Interferon-β (IFNβ) is known to possess robust antitumor effects on different types of cancer cell lines *in vitro*. The present study was aimed to investigate the anti-tumor effect of IFNβ gene-transfected hUCMSCs (IFNβ-hUCMSCs) on breast cancer cells with emphasis on triple negative breast carcinoma. Our findings revealed that the co-culture of IFNβ-hUCMSCs with the human triple negative breast carcinoma cell lines MDA-MB-231 or Hs578T significantly inhibited growth of both carcinoma cells. In addition, the culture medium conditioned by these cells also significantly suppressed the growth and induced apoptosis of both carcinoma cells. Further investigation showed that the suppressed growth and the apoptosis induced by co-culture of IFNβ-hUCMSCs or conditioned medium were abolished by pretreating anti-IFNβ neutralizing antibody. These findings indicate that IFNβ-hUCMSCs triggered cell death of breast carcinoma cells through IFN-β production, thereby induced apoptosis and suppressed tumor cell growth. In conclusion, we demonstrated that IFNβ-hUCMSCs inhibited the growth of breast cancer cells through apoptosis. with potent anti-cancer activity, it represents as an anti-cancer cytotherapeutic modality against breast cancer.

## INTRODUCTION

Breast cancer is the commonest malignancy in most countries in Asia, and the incidence is increasing at a more rapid rate than in western countries, which may be due to changes in the lifestyle and diet [1, 2]. The most major risk factors for breast cancer are known to include hormone replacement therapy such as prolonged exposure to estrogen and/or progesterone and reproductive history [3]. Besides, the importance of estrogen in breast cancer development is also supported by studies demonstrating the occurrence of great changes in estrogen signaling and in the expression of estrogen receptors (ERs), ER alpha and ER beta, during breast carcinogenesis and progression [4–6]. Nowadays, surgical resection followed by radiotherapy and/or chemotherapy is still the most common treatments for breast cancer to prevent its metastasis and recurrence [7]. Therefore, novel treatment strategies for breast cancer are needed.

Interferon-β (IFNβ) has been reported to exhibit robustly inhibitory effects on tumor cell growth and induce apoptosis *in vitro* [8–10]. However, IFNβ gene therapy has not been successfully used in *in vivo* because of its short half-life, as well as the maximally tolerated dose is lower than the effective dose. Although viral vector-based gene delivery is not cancer tissue-specific, several studies have demonstrated that IFNβ gene therapy using adenoviral vectors is effective in several cancers such as ovarian cancer [11], bladder cancer [12], glioma [13], and lung cancer [14]. In addition, the effectiveness of adenoviral vector-based gene delivery to tumor tissues remains unclear. Actually, intratumoral injection of virus vectors showed restricted target protein expression in the cells adjacent to the injection site [15]. To conquer the problem, human bone marrow-derived mesenchymal stem cells (MSCs) have been utilized as biological vehicles for IFNβ gene delivery. This MSC-based IFNβ therapy using systemic administration has been shown to be effective in suppressing metastasis of breast cancer, melanoma [16] and glioma [17, 18].

MSCs derived from the human umbilical cord matrix (hUCMSCs), human postnatal stem cells, possess beneficial properties for therapeutic uses including relatively large cell number of harvest, propagated without any feeder cells, and stored after birth without significant risks to the donor. Rachakatla *et al*. also reported that no teratomas were formed in the SCID mice with injection of hUCMSCs [19]. In addition systemically administered IFNβ gene transfected hUCMSCs (IFNβ-hUCMSCs) is capable of migrating to tumor sites and attenuating growth of breast tumor metastasized to breast [19]. These findings indicate that hUCMSCs can be potential biological vehicles for tumor-targeted delivery of therapeutic agents or genes. However, since this novel therapy has never been applied to the most difficult cancers such as triple negative breast cancer, the aim of this study was to evaluate the therapeutic potential of the IFNβ-hUCMSCs for treating triple negative breast carcinomas. Our findings demonstrated that hUCMSCs producing IFNβ were capable of inhibiting growth of human triple negative breast cancer cells via both extrinsic and intrinsic apoptotic pathways.

## RESULTS

### IFNβ-hUCMSCs significantly inhibited cell growth of MDA-MB-231 and Hs578T cells

**Figure 1:**
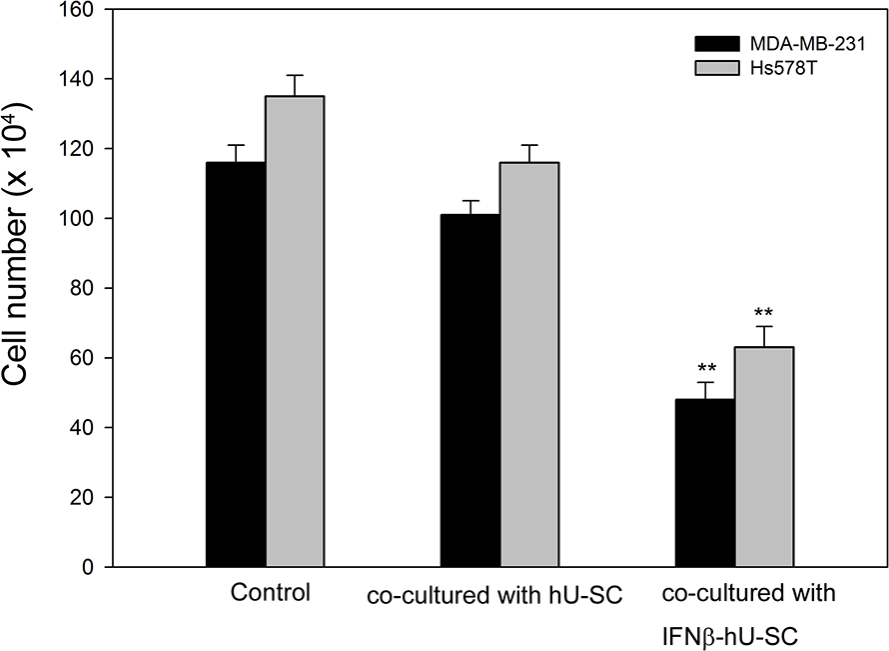
Effect of hUCMSCs and IFNβ-hUCMSCs co-culture on growth of MDA-MB-231 and Hs578T Cells (3 × 10^5^ cells/well) were seeded in 6-well plates and allowed to attach to culture dishes. hUCMSCs or IFNβ-hUCMSCs (3 × 10^5^ cells) were seeded in the cell culture inserts (3.0 μm pore size). The cells were cultured with hUCMSCs growth medium for 72 h, and the cell number was counted using a hemocytometer. Data were presented as means ± SD of the three independent experiments. **P < 0.01 as compared to negative control.

To evaluate the effect of IFNβ-hUCMSCs on cancer cell proliferation, MDA-MB-231 and Hs578T human triple negative breast cancer cells were individually cultured in the bottom of Transwell culture dishes and either hUCMSCs or IFNβ-hUCMSCs were co-cultured in the inserts. After 72 h incubation, numbers of live cells in the bottom of culture dishes were counted. The results revealed that live cell numbers of both MDA-MB-231 and Hs578T cells were significantly decreased after the co-culture with IFNβ-hUCMSCs (Figure 1). In parallel, live cell numbers in both cell lines co-culture with vehicle hUCMSCs were also decreased; however, the decrease of live cell numbers of the tumor cells co-culture with vehicle hUCMSCs was insignificant as compared to tumor cells alone.

### IFNβ in the conditioned medium derived from IFNβ-hUCMSCs reduced cell growth of MDA-MB-231 and Hs578T

**Figure 2:**
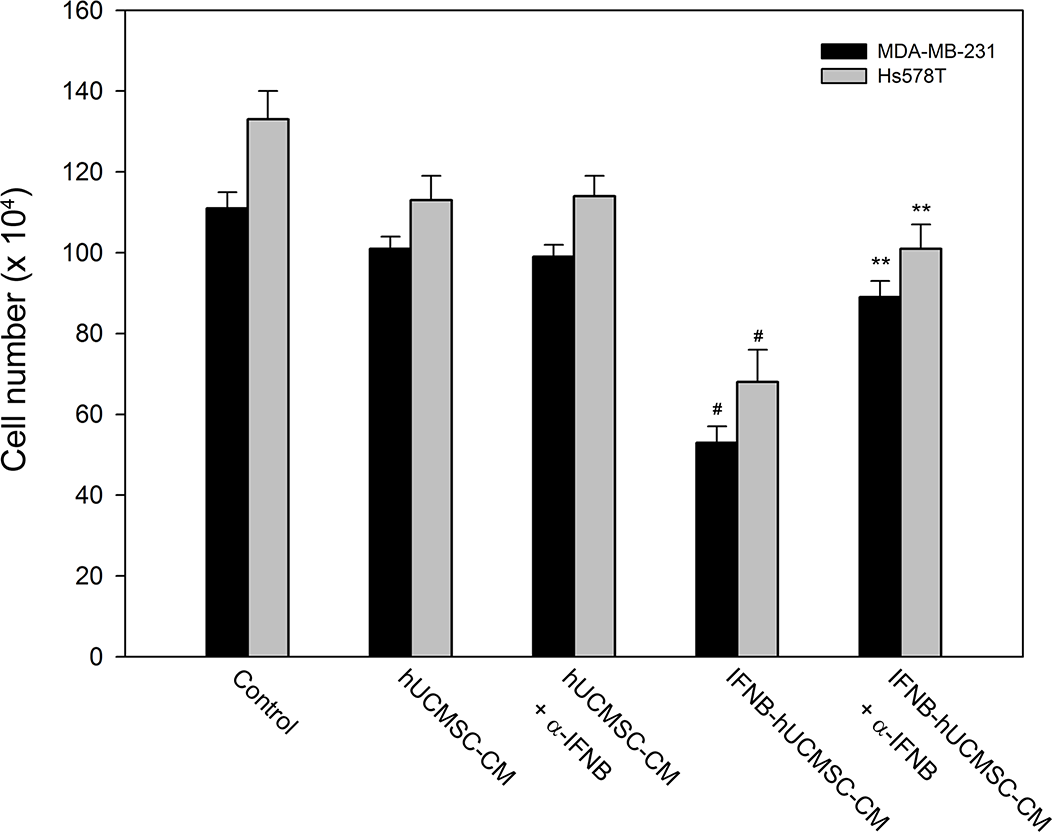
Effect of hUCMSCs or IFNβ-hUCMSCs conditioned medium with or without IFNβ neutralizing antibody on growth of MDA-MB-231 and Hs578T Cells (3 × 10^5^ cells/well) were seeded in 6-well plates and allowed to attach for 24 h. After removing culture medium, the cells were incubated with the hUCMSCs or IFNβ-hUCMSCs conditioned medium with or without IFNβ neutralizing antibody at 5.0 μg/mL for 72 h, and then the cell number was counted using a hemocytometer. Data were presented as means ± SD of the three independent experiments. **P < 0.01 as compared to negative control. ^#^*P* < 0.05 as compared to IFNβ-hU-SC conditioned medium.

Since co-culture with IFNβ-hUCMSCs reduced growth of breast cancer cells, the role of IFNβ in the reduced cell growth was further investigated. As shown in Figure 2, incubation with medium conditioned with IFNβ-hUCMSCs significantly suppressed growth of both MDA-MB-231 and Hs578T cell lines, and the cell growth was lowered down to 53.6 ± 3.7% and 51.1 ± 6.3% of control, respectively. Besides, pre-addition of anti-human IFNβ monoclonal antibody to the conditioned medium neutralized the suppression of cell growth by the IFNβ-hUCMSCs conditioned medium (Figure 2). In parallel, although incubation with medium conditioned with vehicle hU-SC appeared to reduce live cell number of both tumor cell lines, the decrease of the live cell number was insignificant as compared to incubation with control medium. In addition, the pre-addition of anti-human IFNβ monoclonal antibody to the conditioned medium also revealed the similar effects on both tumor cell lines as the conditioned medium alone. Collectively, these observations showed that IFNβ released in IFNβ-hUCMSCs conditioned medium suppressed the growth of both breast tumor cells

### Medium conditioned with IFNβ-hUCMSCs induced apoptosis of MDA-MB-231 and Hs578T cells

**Figure 3:**
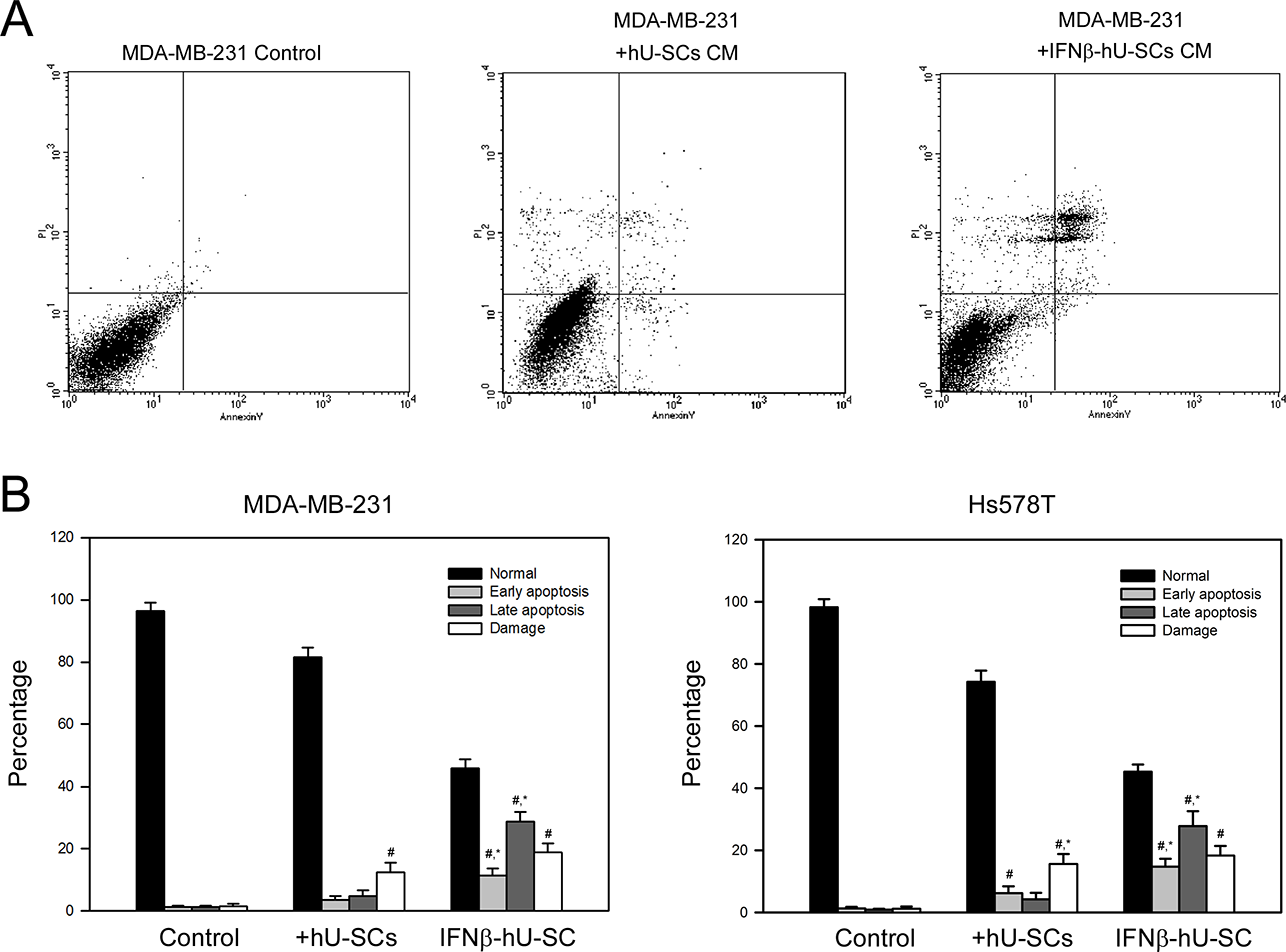
Effect of hUCMSCs or IFNβ-hUCMSCs conditioned medium on the induction of apoptosis in MDA-MB-231 and Hs578T Cells (3 × 10^5^ cells/well) were seeded in 6-well plates and allowed to attach for 24 h. After removing culture medium, the cells were incubated with the hUCMSCs or IFNβ-hUCMSCs conditioned medium for 24 h. The cells were collected, fixed and reacted with PI and Annexin V-FITC, and then subjected to flow cytometric analysis. (**A**) Representative flow cytometric analysis. (**B**) Quantitation for the flow cytometric analysis. PI/Annexin-FITC: −/−, normal; PI/Annexin-FITC: −/+, early apoptosis; PI/Annexin-FITC: +/+, late apoptotic (late); PI/Annexin-FITC = +/−, damaged cells. Data were presented as means ± SD of the three independent experiments. ^#^*P* < 0.05 as compared to control. *P < 0.05 as compared to hUCMSCs conditioned medium.

To investigate whether apoptosis was involved in the inhibited cell growth by IFNβ-hUCMSCs conditioned medium, flow cytometric analysis using PI-Annexin V staining was performed. As shown in Figure 3, culture with the conditioned medium derived from IFNβ-hUCMSCs for 24 h significantly increased percentage of apoptotic cells (early and late apoptosis) for both MDA-MB-231 and H358 cells up to 41.5 ± 3.2% and 43.6 ± 4.1%, respectively. In addition, apoptosis was observed as early as in 6 h after incubation with the conditioned medium (data not shown).

**Figure 4:**
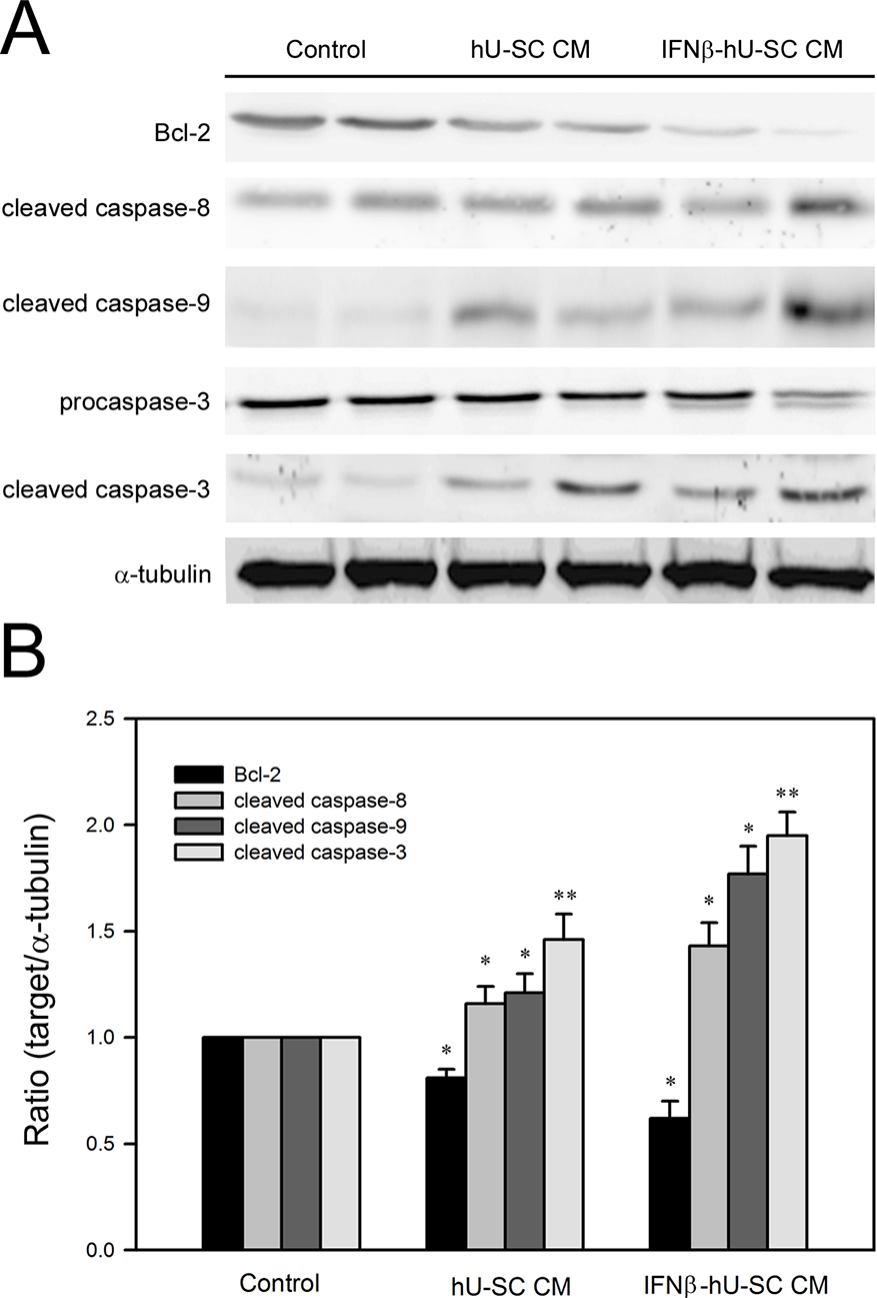
Effect of hUCMSCs or IFNβ-hUCMSCs conditioned medium on caspase cascades in MDA-MB-231 and Hs578T Cells (1 × 10^6^ cells) were seeded in culture plates and allowed to attach for 24 h. After removing culture medium, the cells were incubated with the hUCMSCs or IFNβ-hUCMSCs conditioned medium for 48 h. (**A**) The cells were collected and lysed for immunodetection of Bcl-2 and caspases using specific antibodies. (**B**) Quantitative data were acquired using densitometric analysis from three independent experiments. * and **p < 0.05 and *p* < 0.01 as compared to negative control.

The apoptosis induced by IFNβ-hUCMSCs conditioned medium was also evaluated by immunoblotting analysis of caspase cascades. Consistent with flow cytometry analysis, the protein levels of cleaved caspase-8, cleaved caspase-9 and cleaved caspase-3 were increased in response to IFNβ-hUCMSCs conditioned medium (Figure 4). Furthermore, culture with IFNβ-hUCMSCs conditioned medium contributed to decrease of anti-apoptotic Bcl-2 level (Figure 4). Together, these results showed that IFNβ-hUCMSCs conditioned medium significantly induced cell apoptosis and triggered activation of caspase cascades in the breast tumor cells.

### Involvement of JAK/STAT signaling in medium conditioned with IFNβ-hUCMSCs induced apoptosis of MDA-MB-231

**Figure 5:**
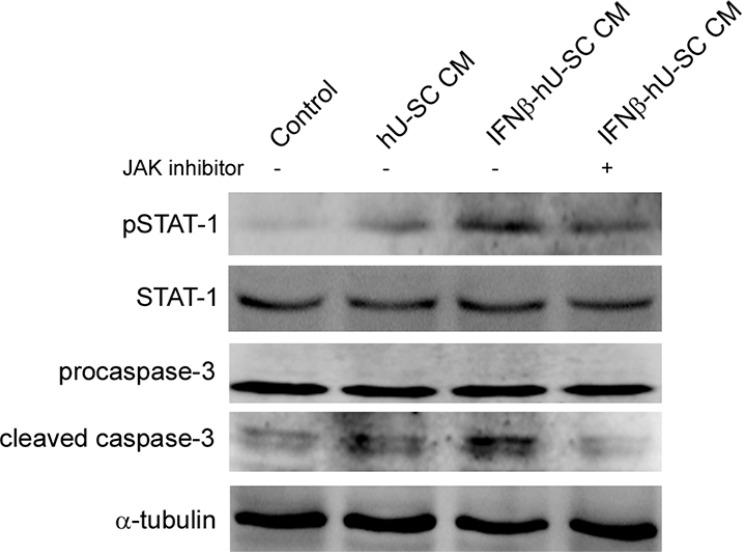
Involvement of Jak-Stat pathway in IFNβ-hUCMSCs conditioned medium induced apoptosis MDA-MB-231 cells were pretreated with JAK inhibitor (5 μM) for 2 h and then treated with indicated conditioned medium for 48 h. The treated cells were collected and lysed for immunodetection of pSTAT-1, STAT-1 and caspase-3. α-tubulin was used as internal control.

JAK/STAT signaling is known to play an important role in type I interferon induced apoptosis. Therefore, the involvement of JAK/STAT signaling in IFNβ- hUCMSCs induced apoptosis was then investigated. As shown in Figure 5, culture with the conditioned medium derived from IFNβ- hUCMSCs for 24 h significantly increased phosphorylation of STAT-1 (pSTAT-1) in MDA-MB-231 cells. In addition, inhibition of JAK reduced both pSTAT-1 and cleavage of procaspase-3. These results revealed that JAK/STAT signaling involved in IFNβ- hUCMSCs induced apoptosis.

## DISCUSSION

Mounting evidences suggest that endogenous apoptosis inducers and cell growth regulators are important targets for effective cancer therapy [23–25]. Recently, a number of such gene products and inhibitors for growth factors are increasingly tested in clinical trials [26, 27]. IFN-β is a cytokine that robustly induces caspase-mediated apoptosis of cancer cells through activating different pathways such as mitochondrial and Jak-Stat signaling [28, 29]. IFN-β is also a potent inhibitor of proliferation of many cancer cell lines *in vitro* [30]. However, use of IFNβ for cancer therapy is difficult due to its short half-life, as well as due to the low systemic tolerated dose that is inefficient to exert the apoptosis-inducing effects [31, 32]. Studeny *et al*. [16] has shown that the local production of IFNβ in tumor tissues plays an important role in IFNβ-based cancer therapy. Thus, stem cell-based therapeutic gene delivery to tumor tissues appears to be a promising approach for local production of therapeutic cytokine and avoiding the adverse effects of systemic cytotoxic cytokines. Similarly, our present study indicates that hUCMSCs based IFNβ gene delivery effectively suppresses growth of triple negative breast cancer cells, which is one of the most difficult cancers to treat. Further studies *in vivo* are required to demonstrate the homing property of IFNβ-hUCMSCs to breast tumor tissue.

Aberrant cells such as mutated or proliferating neoplastic cells are removed by programmed cell death, namely apoptosis [33]. Two well-known pathways, extrinsic and intrinsic pathways, are responsible for triggering apoptosis [34]. For the growth inhibition by IFNβ, our results revealed that IFNβ-hUCMSCs conditioned medium significantly increased Annexin V-FITC positive cells and the increase of Annexin V-FITC positive cells was diminished by pretreating with IFNβ neutralizing antibody, indicating IFNβ production plays a central role in the induction of apoptosis. Moreover, these findings also demonstrate that IFNβ-hUCMSCs C is an efficient gene delivery vector that produces enough IFNβ to induce apoptosis and the consequent cell growth inhibition. Consistent to increase of Annexin V-FITC positive cells, the immunoblotting analysis showed that IFNβ-hUCMSCs conditioned medium significantly triggered activation of caspase-8, caspase-9 and caspase-3. Taken together, these findings indicate that the IFNβ-dependent cell growth attenuation attributes to the activation of both extrinsic and intrinsic apoptosis pathways. To the best of our knowledge, this is the first demonstration of the efficacy of IFNβ gene transfected stem cell therapy on breast carcinomas, proposing that IFNβ-hUCMSCs is a potential therapeutic agent for breast cancer. Nevertheless, the effective dose of IFNβ-hUCMSCs as well as working concentration of IFN-β in preclinical and clinical setting requires further studies.

Regarding tumor tissue-targeted homing of hUCMSCs, interactions between chemokines produced by tumor tissues and receptor expression in stem cells have been suggested. Various chemokines can be secreted by tumors, including vascular endothelial growth factor (VEGF), Transforming growth factor (TGF) family members, fibroblast growth factor (FGF) family members, Platelet-derived growth factor (PDGF) family members, MCP-1, EGF, and IL-8 [35]. Interestingly, the bone marrow MSCs exhibit a tropism for damaged or rapidly growing tissues as well as tumors [36, 37]. Previous study also indicates that hUCMSCs home to tumor tissues but not to healthy tissues and express multiple chemokine receptors, such as SDFR1, TGFBR3, FGFR2 [19, 38]. Thus, it is conceivable that IFNβ-hUCMSCs administered via the tail vein exhibited a selective from the tumor and/or tumor-associated cells. These properties of hUCMSCs encourage their development as a therapeutic agent for tumors.

## MATERIALS AND METHODS

### Reagents

RPMI-1640, L-15 medium, fetal bovine serum (FBS), low glucose Dulbecco's modified Eagle's medium (DMEM), insulin-transferrin-selenium-X (ITS-X), and ALBUMax1 were purchased from Invitrogen (Carlsbad, CA, USA). MCBD 201 medium, ascorbic acid 2-phosphate, and dexamethasone were from Sigma-Aldrich (St. Louis, MO, USA). Epidermal growth factor (EGF) and platelet derived growth factor-BB (PDGF-BB) were from R & D Systems (Minneapolis, MN, USA).

### Cell culture

The breast cancer cell line MDA-MB-231 and Hs578T was obtained from American Type Culture Collection (ATCC; Rockville, MD, USA) and maintained in DMEM supplemented with 10% v/v fetal bovine serum, 1% nonessential amino acid, 1% L-glutamine (Gibco BRL, Gaithersburg, MD, USA) and 100 μg/mL penicillin/streptomycin (Sigma) at 37°C in a humidified atmosphere with 5% CO_2_. hUCMSCs were prepared from human umbilical cord Wharton's jelly obtained from Taipei Medical University with Institutional Review Board guidance. Culture of hUCMSCs was performed as previously described [19]. The culture medium for hU-SC was low glucose DMEM containing 37% MCDB 201, 2% FBS, 1% ITS-X, 1.5 g/mL ALBUMax1, 10 nM dexamethasone, 50 μM ascorbic acid 2-phosphate, 1 ng/mL EGF, 10 ng/mL PDGF-BB, 100 units/mL penicillin and 100 μg/mL streptomycin. The cells were incubated in 5% CO_2_ humidified air at 37°C.

### Gene transduction with adenoviral vectors

The RGD fiber-modified adenoviral vectors encoding genes for human IFNβ were prepared as previously described [20, 21], and the gene transduction to hUCMSCs was performed according to the previous procedure [19]. Twenty four hours (h) after gene transduction, the IFNβ-hUCMSCs were used for the following experiments.

### Co-culture of hUCMSCs and IFNβ-hUCMSCs with breast cancer cells

MDA-MB-231 or Hs578T were maintained in normal culture media at 4 × 10^5^ cells per well in 6-well plates. Prior to experiments, 3 × 10^5^ hUCMSCs or IFNβ-hUCMSCs were seeded into Transwell cell culture inserts (3.0 μm pore size, BD Biosciences, San Jose, CA, USA) and cultured with the growth medium for hUCMSCs for 72 h. The cancer cell cultures were lifted, and the number of the cancer cells was counted using a hemocytometer.

### Culture with hUCMSCs and IFNβ-hUCMSCs conditioned media

The normal culture media were conditioned by culturing IFNβ-hUCMSCs or hUCMSCs for 24 h. The conditioned media were used for treatment of the breast cancer cell lines. Neutralizing antibody against IFNβ (mouse anti-human IFNβ monoclonal antibody, Chemicon, Temecula, CA, USA) was added to the conditioned media at a final concentration of 5 μg/mL. The viable cancer cell number was counted after 72 h of culture.

### Flow cytometric analysis for apoptotic cells

The percentage of apoptotic cells was determined using an Annexin V-FITC apoptosis detection kit (Biovision, Mountain View, CA, USA). Briefly, cells were washed with cold PBS and re-suspended in binding buffer (10 mM HEPES, pH 7.4, 140 mM NaCl and 2.5 mM CaCl_2_) at a concentration of 5–10 × 10^6^ cells/mL. Cells were incubated at room temperature with 5 μL each of Annexin V-fluorescein isothiocyanate (FITC) and propidium iodide (PI) for 5 minutes, and analyzed on a with a FACS Calibur system (version 2.0, BD Biosciences, Franklin Lakes, NJ, USA) and CellQuest software.

### Immunoblotting analysis

Crude proteins were prepared using lysis buffer (1% Triton X-100, 0.1% SDS, 0.25 M sucrose, 1 mM EDTA, 30 mM Tris-HCl, pH 8.0) supplemented with protease inhibitor cocktail (Boehringer Mannheim, Indianapolis, IN), and subjected to a 12.5% SDS-polyacrylamide gel and transferred onto a nitrocellulose membrane as previously described [22]. The blot was subsequently incubated with 5% nonfat milk in PBS for 1 h, probed with a 1/1000 dilution of primary antibodies against Bcl-2, caspase-3, caspase-8, caspase-9, phospho-STAT-1 (pSTAT-1) and STAT-1 (Cell Signaling Technology, Beverly, MA, USA), or α-tubulin (α-TN, Santa Cruz Biotechnology, Santa Cruz, CA, USA) for 2 h, and then reacted with 1/2000 dilution of peroxidase-conjugated secondary antibody for 1 h. All incubations were carried out at 30^°^C, and intensive PBS washing was performed between each incubation. After the final PBS wash, the signal was developed by ECL chemiluminescence, and the relative photographic density was quantitated by image analysis system (Fuji Film, Tokyo, Japan).

### Statistical analysis

Data were expressed as means ± SD of the three independent experiments. Statistical significance analysis was determined by using one-way ANOVA followed by Dunnett for multiple comparisons with the control. The differences were considered significant for *p* values less than 0.05.
